# The Pattern of Cervical Cancer according to HIV Status in Yaoundé, Cameroon

**DOI:** 10.1155/2021/1999189

**Published:** 2021-10-13

**Authors:** Pierre-Marie Tebeu, Jean Pierre Ngou-Mve-Ngou, Laure Leka Zingué, Jesse Saint Saba Antaon, Etienne Okobalemba Atenguena, Julius Sama Dohbit

**Affiliations:** ^1^Department of Obstetrics and Gynaecology, Faculty of Medicine and Biomedical Sciences, University of Yaoundé I, Yaoundé, Cameroon; ^2^League of Initiative and Active Research for Women's Health and Education (LIRASEF), Yaoundé, Cameroon; ^3^Inters-states Centre for Public Health Training in Central Africa, Brazzaville, Congo; ^4^Department of Obstetrics & Gynecology, University Hospitals, Libreville, Gabon; ^5^Department of Internal Medicine, Faculty of Medicine and Biomedical Sciences, University of Yaounde I, Yaoundé, Cameroon

## Abstract

**Objective:**

To analyze the epidemiological aspects of invasive cervical cancer according to HIV status.

**Methods:**

This was an historical cohort study from January 2010 to April 2017 in three hospitals at the Yaoundé city Capital, Cameroon, after the National Ethics Committee' approval. We included invasive cervical cancers with documented HIV status. Odds ratios and 95% confidence interval were calculated to assess the association between the different variables and HIV status. Survival was analyzed using the Kaplan–Meier. The level of significance was set up at <5%.

**Results:**

Among the overall 213 cervical cancer patients, 56 were HIV+ (24.67%). Factors associated with positive HIV status were age below 40 (OR: 2.03 (1.38–2.67)), celibacy (OR: 2.88 (1.58–4.17)), nonmenopausal status (OR: 2.56 (1.36–3.75)), low parity, primiparity (OR: 2.59 (1.43–3.74)), and for parity with 2–4 children (OR: 2.24 (1.35–3.12)). Concerning the HIV+ patients, tumor was diagnosed late (stages III-IV) (OR: 2.70 (1.43–5.08)), undifferentiated (grade III) (OR: 7.69 (5.80–9.57)), with low median survival (9.83 months vs. 20.10 months).

**Conclusion:**

HIV is frequent among cervical cancer patients. In the HIV+ patients, the diagnosis was made at the advanced stage, cells were poorly differentiated, and the prognosis was worse.

## 1. Introduction

Cervical cancer is a malignant neoformation developed at the opening of the cervix, most often on the exocervical mucosa [1]. Cervical cancer starts by a precancerous lesion known as benign and asymptomatic epithelial abnormality. Proper management of these precancerous lesions prevents development towards invasive cancer [1, 2].

In resource-limited countries, cervical cancer is the second leading cause of death from cancer in women, after breast cancer [1]. This sexually transmitted disease progresses very slowly between 10 and 20 years from infection to premalignant and malignant lesion giving a great possibility of screening [2]. In 2018, GLOBOCAN estimated that there were more than 570,000 new cases diagnosed a year, and 311,000 women die during the same period [3]. GLOBOCAN also estimated that there were more than 32,000 new cases of cervical cancer in Central Africa, with about 23,000 deaths during the same year [3].

There is a wide geographical disparity in the distribution of cervical cancer worldwide, with over 85% of cases in developing countries [3]. This disparity in geographical distribution of cervical cancer, with high morbidity and mortality in developing countries, is mainly due to the absence of systematic screening for this cancer and comorbidity with the advent of HIV/AIDS pandemic in these poor regions [3, 4].

HIV infection is also a real public health issue worldwide and mainly in African countries leading to critical consequences, including morbidity and mortality [4]. HIV infection generally increases the risk of cancer and mainly the viral-induced one [4]. Many studies documented that HIV infection and the subsequent immunodeficiency are associated with increased risk of precancerous lesions [4–6]. Therefore, HIV-infected women are 4–9 times more likely to develop cervical cancer [7, 8]. In 1993, the revised classification by CDC (Centre of Disease's Control and Prevention), included the invasive carcinoma of the cervix among the diseases that move from HIV's infection stage to AIDS disease one [9].

In Cameroon, cervical cancer is the second cancer in women, just after breast cancer (25.2% vs. 35.1%) [3]. Moreover, in some series, cervical cancer stands before breast cancer [10, 11]. In the same country, HIV has a prevalence of 1.9% in men aged 15–64 and 3.6% in women of the same age group [12].

Little is known about the coexistence of the two diseases in Cameroon.

### 1.1. Objective

To analyze the epidemiological aspects of invasive cervical cancer according to HIV status in Yaoundé, Cameroon.

## 2. Materials and Methods

### 2.1. Type–Place–Period

This is an historical cohort study over a 7-year period (January 2010–April 2017) in Yaoundé, Cameroon. The three study sites were Yaoundé University Hospital Centre (YUTH), Yaoundé General Hospital (YGH), and Yaounde Gynaeco-Obstetric and Paediatric Hospital (YGOPH). The study was approved by these hospitals.

### 2.2. Study Population

The study population included women in whom invasive cervical cancer was detected. After obtaining their verbal consent, all patients with invasive cervical cancer with documented histological diagnosis and HIV status were included.

### 2.3. Variables

Data on HIV status, sociodemographic, reproductive, clinical, and therapeutic characteristics of patients were collected. Data collection was done from medical records and phone calls after verbal consent.

### 2.4. Data Analysis

The data were analyzed using Epi-Info 7.2.2.6 software (CDC, USA) and SPSS 20.0 software (IBM, Armonk, NY, USA). The absolute and relative frequencies as well as central tendency (mean and median) and dispersions parameters were calculated. The odds ratios were calculated within 95% confidence interval in order to assess the influence between the different variables and HIV status. The dependent variables considered were the HIV status of patients with invasive cervical cancer and the survival period. Pearson, Fisher, and Wald statistical tests were used. The level of significance was set up at *p* < 5%.

## 3. Results

A total of 213 invasive cervical cancer patients were recorded, including 56 with HIV+ status (24.67%). The median age of invasive cervical cancer patients was 12 years lower for HIV+ patients compared to their HIV− status counterparts ((43 vs. 55 years); *p* < 0.001).

HIV+ patients were frequently received at advanced cancer stage (FIGO III-IV (57, 15% vs. 32.48%)). Squamous cell carcinoma was the histological type mostly found in both groups (87.5% HIV+ status vs. 89.81% for HIV− one). Cancer in HIV+ women was mostly with undifferentiated cells (35.7% vs. 3.82%).

Younger cervical cancer patients (<40 years old) were more likely to be HIV+ than elder ones (40–60 years) (54.7% vs. 28.9%; OR: 2.03 (1.38–2.67); *p*=0.017). Single/divorced cervical cancer women were more at risk of being HIV+ than married ones (46.6% vs. 16.1%, OR: 2.88 (1.58–4.17), *p*=0.037; 16.1% vs. 50%, OR: 3.08 (1.69–4.46), *p*=0.023) (Table 1).

Premenopausal women were 2.56 times more likely to be HIV+ than postmenopausal ones (17.14% vs. 43.84%, OR: 2.56 (1.36–3.75), *p*=0.016).

Lower/mild parity women (<5 children) had more risk of being HIV+ than high parity ones (58.82% vs. 84.10%, OR: 2.59 (1.43–3.74), *p*=0.0016 and 56.25% vs. 84.10%, OR: 2.59 (1.43–3.74), *p*=0.001).

Women with more than five cumulative sexual partners were more at risk of being HIV+ (21.57% vs. 48.39%, OR: 2.24 (1.35–3.12), *p*=0.005) (Table 2).

FIGO stages III-IV cervical cancer patients were more likely to be HIV+ status than those of stages I-II (40.51% vs. 18.85%, OR: 2.70 (1.43–5.08), *p*=0.001).

Compared to patients with good differentiation cell (grade I), patients with poor differentiation (grades II and III) had more risk of being HIV+ status (10% vs. 41.94% OR: 4.19 (3.16–5.21), *p*=0.041) and (10% vs. 72.96%, OR = 7.69 (5.80–9.57), *p*=0.005) (Table 3).

**Figure 1:**
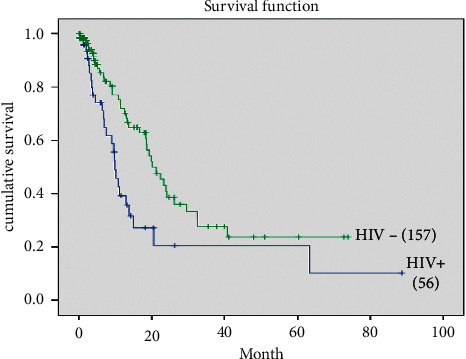
Survival curve according to HIV status.

HIV+ cervical cancer patients had a median survival lower than the HIV− patients (9.83 months; 95% CI (8.32–11.3) vs. 20.10 months; 95% CI (15.33–24.9)) (Figure 1).

## 4. Discussion

We found an HIV frequency of 24.67% in cases of invasive cervical cancer; therefore, about 1 out of 4 women was HIV+. In Cameroon, the average frequency of HIV infection (reported by the 2018 Demographic Health Survey) is 2.9%, with some gender disparities: 1.9% in men and 3.6% in women [12]. The high frequency of HIV-positive status among patients with invasive cervical cancer compared to HIV prevalence in the general population in women may be due to the fact that HIV infection increases the risk of invasive cervical cancer. Several studies have analyzed the HIV prevalence in invasive cervical cancer patients. Some findings are similar to ours, with proportions of 21 and 25% [13, 14]. Others found proportions higher than ours (29.7–66.4%) [15–20]. A study in Botswana reported a prevalence of 66.4% [18]. These differences may be due to the fact that HIV prevalence in women in Cameroon (3.6% according to the latest Demography Health Survey) is lower than HIV prevalence in Botswana (23.4% in 2013, according to the UNAIDS report) [12, 21].

As for the factors associated with HIV+ status, we found that, compared to older women (40–60 years), younger ones (<40 years) had twice the risk of being HIV+ (OR: 2.03 (1.38–2.67)). Our findings may be due to the fact that HIV infection accelerates the development of precancerous lesions to invasive cancer. These findings corroborate those of many other authors related to the young age of HIV+ patients with cervical cancer at diagnosis. A study in Tanzania revealed findings similar to ours. They found that young patients with invasive cervical cancer under 50 years were more likely to be HIV+ [20].

Single women were more likely to be HIV+ among ICC patients (OR: 2.88 (1.58–4.17)). Similarly, separated/divorced women were more likely to have increased risk of being HIV+ (OR: 3.08 (1.69–4.46)). These findings may be partly due to the fact that people not involved in a stable exclusive relation are much more exposed to HIV. This report on separated/divorced status is similar to that of the 2011 DHS-MICS; HIV prevalence was highest in widows (17.9%) and divorcees (15.7%) [22].

Compared to patients living in rural areas, those living in urban ones were more likely to be HIV+ (OR: 3.66; 95% CI: 1.14–6.17). These findings corroborate those of the 2018 HDS-MICS, which reported the higher HIV prevalence in women living in urban areas compared to those living in rural ones (3.9% vs. 2.9%) [12].

As for parity, our findings revealed that women of low parity were more likely to be HIV+. These findings may be due to the fact that HIV+ status could decrease the desire for maternity in some women. This could also be due to the fact that ICC occurs more in HIV+ women at a younger age [8].

Women with a history of cumulated sexual partners (CSP) ≥ 5 were more likely to be HIV+, compared to those with less than 5 CSP (OR: 2.24 (1.35–3.12)). These findings may be due to the fact that a history of many sexual partners is a common risk factor for HIV and ICC [2]. These findings are similar to those of a study in Tanzania that reported a history of many sexual partners as a factor associated with HIV+ status (>6) (OR: 5.56; 95% CI: 1.18–26.25) [20].

HIV+ patients mostly came with stages III-IV and grades II and III (57.15%). Other authors highlighted the association between HIV and poorly differentiated tumor cells [23]. These findings are similar to those in Tanzania that found the possibility six times greater to be HIV+ in women with tumor poorly differentiated cells than those with good or moderate differentiated ones [24].

In HIV+ women, the median of survival was worse than in HIV− ones (9.83 months vs. 20.10 months). Therefore, half of HIV+ patient with ICC died in the same year, before the tenth month; half of HIV− ones died before the second year, at least during the first 20 months. Lower survival rates in HIV+ patients with ICC compared to HIV− ones were underlined by other authors [24–27]. These results may be due to the advanced stage at diagnosis and poor cell differentiation in HIV+ patients [24]. HIV+ patients with ICC could die sooner of opportunistic infections probably due to the deteriorating of immune-suppression condition. Our findings are similar to those of Dryden Peterson et al., in 2016 in Botswana, who also found that HIV+ patients had a shorter median survival than HIV− ones (21.7 months vs. 30.5 months), i.e., a 9-month difference between the 2 groups [18].

Median's survival of HIV+ and HIV− found in our study are 12 months lower than those found in Botswana. This significant difference may be due to different therapeutic modalities in each of the two countries, grades disparity of tumoral differentiation and treatment compliance.

## 5. Conclusion

HIV infection prevalence is high in patients with ICC (24.67%). HIV+ patients with ICC are younger, most often received advanced stages III-IV, and have mostly poorly differentiated cells. HIV+ patients with ICC have survival median lower than HIV− ones. We emphasize on the need to create multidisciplinary care units for people living with HIV+ including cervical cancer prevention.

## Figures and Tables

**Table 1:** Distribution of the sociodemographic characteristics of ICC patients according to the HIV status in bivariate analysis.

Variables	HIV status			OR (95% CI)	*P* value
	Total *N* = 213	HIV+ *N* = 56 *n* (%)	HIV− *N* = 157 *n* (%)		
Age at diagnosis of ICC					
<40	42	23 (54.7)	19 (45.24)	2.03 (1.38–2.67)	0.017
40–60	114	33 (28.9)	81 (71.05)	1^a^	
>60	57	0 (0)	57 (100)	0	0.996

Profession					
Employee/self-employment	47	14 (29.7)	33 (70.21)	1.35 (0.19–2.5)	0.997
Official	22	6 (27.27)	16 (72.73)	1.24 (0.18–2.29)	0.653
Unemployed	127	28 (22.0)	99 (77.95)	1	
Not specified	17	8 (47.06)	9 (52.94)		

Educational level					
Primary	67	10 (14.9)	57 (85.07)	1	
Secondary	90	33 (36.6)	57 (63.33)	2.46 (0.95–3.96)	0.227
Superior	13	2 (15.38)	11 (84.62)	1.03 (0.40–1.65)	0.077
Not specified	43	11 (25.58)	32 (74.42)		

Marital status					
Single	45	21 (46.6)	24 (54.33)	2.88 (1.58–4.17)	0.037
Married	105	17 (16.1)	88 (83.80)	1	
Separated/divorced	4	2 (50)	2 (50)	3.08 (1.69–4.46)	0.023
Widow	48	11 (22.9)	37 (77.09)	1.41 (0.77–2.04)	0.177
Not specified	11	5 (45.45)	6 (54.55)		

Place of residence					
Urban	148	45 (30.4)	103 (69.59)	3.66 (1.14–6.17)	0.029
Rural	36	3 (8.33)	33 (91.67)	1	
Not specified	29	8 (27.59)	21 (72.41)		

^a ^Reference category.

**Table 2:** Reproductive characteristics distribution of ICC patients based on HIV status in bivariate analysis.

Variables	HIV status			OR (95% CI)	*P* value
	Total *N* = 213	HIV+ *N* = 56 *n* (%)	HIV− *N* = 157 *n* (%)		
Menopause					
Yes	140	24 (17.14)	116 (82.86)	1^a^	—
No	73	32 (43.84)	41 (56.16)	2.56 (1.36–3.75)	0.016

Parity					
1	17	7 (41.18)	10 (58.82)	2.59 (1.43–3.74)	0.016
2–5	64	28 (43.75)	36 (56.25)	2.75 (1.52–3.97)	0.001
5–14	132	21 (15.9)	111 (84.10)	1	—

Gravidity					
0–5	59	25 (42.37)	34 (57.63)	2.1 (0.85–3.34)	0.194
5–18	154	31 (20.13)	123 (79.87)	1	—

Number of CSP^b^					
1	13	0 (0.0)	13 (100)	0	0.050
2–5	51	11 (21.57)	40 (78.43)	1	—
5–15	62	30 (48.39)	32 (51.61)	2.24 (1.35–3.12)	0.005
Not specified	87	15 (17.24)	72 (82.75)		

Use of contraceptive					
Yes	42	15 (35.71)	27 (64.29)	1.88 (0.59–3.16)	0.679
No	122	23 (18.85)	99 (81.15)	1	—
Not specified	49	18 (36.73)	31 (63.27)		

^a^Reference category. ^b^Cumulative sexual partner.

**Table 3:** Distribution of ICC patients according to FIGO stage, degree of differentiation, and HIV status in bivariate analysis.

Variables	HIV status			OR (95% CI)	*P* value
	Total	HIV+	HIV−		
	*N* = 213	*N* = 56	*N* = 157		
		*n* (%)	*n* (%)		
FIGO stages					
Stage I	57	11 (19.30)	46 (80.7)	1.04 (0.44–1.63)	0.969
Stage II	65	12 (18.46)	53 (81.54)	1^a^	—
Stage III	60	25 (41.67)	35 (58.33)	2.25 (0.96–3.54)	0.421
Stage IV	23	7 (30, 43)	16 (69.57)	1.64 (0.70–2.58)	0.115
Not specified	8	1 (12.5)	7 (87.5)		

Degree of differentiation					
Grade I	50	5 (10)	45 (90)	1	—
Grade II	62	26 (41.94)	36 (58.06)	4.19 (3.16–5.21)	0.041
Grade III	26	20 (72.96)	6 (27.04)	7.69 (5.80–9.57)	0.005
Not specified	75	5 (6.67)	70 (93.33)		

^a^Reference category.

## Data Availability

The data used to support the findings of this study are available from the corresponding author upon request.
